# Subjective cognitive decline symptoms and its association with socio-demographic characteristics and common chronic diseases in the southern Chinese older adults

**DOI:** 10.1186/s12889-022-12522-4

**Published:** 2022-01-18

**Authors:** Li-Hua Lin, Shi-Bin Wang, Wen-Qi Xu, Qing Hu, Ping Zhang, Yun-Fei Ke, Jia-Hao Huang, Kai-Rong Ding, Xue-Li Li, Cai-Lan Hou, Fu-Jun Jia

**Affiliations:** 1 School of Medicine, South China University of Technology, Guangzhou, 510006 Guangdong Province China; 2 Guangdong Mental Health Center, Guangdong Provincial People’s Hospital, Guangdong Academy of Medical Sciences, Guangzhou, 510080 Guangdong Province China; 3 School of Nursing, Southern Medical University, Guangzhou, 510515 Guangdong Province China; 4 Yuexiu District Center for Disease Control, Guangzhou, 510080 Guangdong Province China; 5 Department of Psychology, School of Public Health, Southern Medical University, Guangzhou, 510515 Guangdong Province China

**Keywords:** Chronic diseases, Subjective cognitive decline symptoms, Cross-sectional study, Chinese older adults

## Abstract

**Background:**

Subjective cognitive decline (SCD) may be the first symptomatic manifestation of Alzheimer’s disease, but information on its health correlates is still sparse in Chinese older adults. This study aimed to estimate SCD symptoms and its association with socio-demographic characteristics, common chronic diseases among southern Chinese older adults.

**Methods:**

Participants aged 60 years and older from 7 communities and 2 nursing homes in Guangzhou were recruited and interviewed with standardized assessment tools. Pittsburgh Sleep Quality Index (PSQI), Patient Health Questionnaire–9 (PHQ-9) and Generalized Anxiety Disorder-7 (GAD-7) were used to measure poor sleep quality, depression symptoms and anxiety symptoms. The SCD symptoms were measured by SCD questionnaire 9 (SCD-Q9) which ranged from 0 to 9 points, with a higher score indicating increased severity of the SCD. Participants were divided into low score group (SCD-Q9 score ≤ 3) and higher score group (SCD-Q9 score > 3). Chi-square tests and multivariate logistic regression analysis were used for exploring the influences of different characteristics of socio-demographic and lifestyle factors on SCD symptoms. Univariate and multivariate logistic regression analysis were applied to explore the association between SCD symptoms with common chronic diseases.

**Results:**

A total of 688 participants were included in our analysis with a mean age of 73.79 (*SD* = 8.28, range: 60–101), while 62.4% of the participants were females. The mean score of the SCD-Q9 was 3.81 ± 2.42 in the whole sample. A total of 286 participants (41.6%) were defined as the low score group (≤3 points), while 402 participants (58.4%) were the high score group (> 3 points). Multivariate logistic regression analysis revealed that female (*OR* = 1.99, *95%CI:* 1.35–2.93), primary or lower education level (*OR* = 2.58, *95%CI*: 1.38–4.83), nursing home (*OR* = 1.90, *95%CI*: 1.18–3.05), napping habits (*OR* = 1.59, *95%CI*: 1.06–2.40), urolithiasis (*OR* = 2.72, *95%CI*: 1.15–6.40), gout (*OR* = 2.12, *95%CI*: 1.14–3.93), poor sleep quality (*OR* = 1.93, *95%CI*: 1.38–2.71), depression symptoms (*OR* = 3.01, *95%CI*: 1.70–5.34) and anxiety symptoms (*OR* = 3.11, *95%CI*: 1.29–7.46) were independent positive related to high SCD-Q9 score. On the other hand, tea-drinking habits (*OR* = 0.64, *95%CI*: 0.45–0.92), current smoking (*OR* = 0.46, *95%CI*: 0.24–0.90) were independent negative related to high SCD-Q9 score.

**Conclusions:**

Worse SCD symptoms were closely related to common chronic diseases and socio-demographic characteristics. Disease managers should pay more attention to those factors to early intervention and management for SCD symptoms among southern Chinese older adults.

## Background

Subjective cognitive decline (SCD) was demonstrated as the subjective cognitive decline in those with normal cognition, involving cognitive domains of processing speed, executive functions, memory, and visuospatial abilities, especially in the memory domain [1, 2]. And SCD does not need reconfirm by cognitive tests when its diagnosis is not required [3]. Before redefined in 2014, SCD was studied as subjective cognitive impairment, subjective memory decline, subjective memory impairment, and memory complaint in many studies [4]. A large cohort study found that memory complaints occurred around 16 years before the dementia diagnosis [5]. Compared with those without SCD, older adults with SCD were 2.3 times as likely to develop dementia and mild cognitive decline (MCI) [6]. Similarly, a multicenter study found that those with SCD presented a higher incidence rate of dementia than those without SCD [7]. A phenomenological study describing the cognitive changes of health, MCI and Alzheimer’s disease (AD) in older adults stated that cognitive decline among the three groups showed an increasing relationship [8]. Therefore, SCD was considered as an early marker for impending cognitive decline in older adults. According to a previous survey, the prevalence of SCD was 14.4–18.8% among the northern Chinese population [9]. But there are few studies related to SCD in southern China. Consequently, it is of great public health significance to study SCD symptoms among southern older adults.

China has already been a society of aging, which is increasing the burden of current family and public health care systems and the main cause of extensive chronic diseases linked with poor quality of life in older adults, including in ischemic heart disease, diabetes, neurodegenerative diseases, mental disorders and so on [10–15]. The prevalence of AD, MCI was 3.9 and 15.5% respectively in 2020 and tightly linked to common chronic diseases, involving cardiovascular diseases, diabetes, hyperlipidemia, cerebrovascular diseases among Chinese older adults [16–20]. The increasing prevalence of AD and MCI is becoming an important public health issue in China. But the most commonly used treatment of AD in medical institutions is still symptomatic treatment in present China. It is of paramount importance to prevent and intervene cognitive-related diseases from the perspective of managing chronic diseases.

SCD may be the first symptomatic manifestation of AD and has been proved to be linking to a range of factors including hypertension, diabetes, fatigue, depression symptoms and anxiety symptoms [21–24]. A study found that SCD was associated with higher systemic levels of pro-inflammatory markers MCP-1, IL-6 and CRP, in particular about executive cognitive functioning [25]. Many chronic diseases are related to inflammation. While previous studies mostly focused on memory complaints while ignoring other cognitive domains such as attention, executive function, and the results were also contradictory due to the different measurement methods. Further studies are required to estimate the association between SCD and common chronic diseases.

Information on SCD is relatively scarce in China. A review reporting the measurements of SCD used in 19 international studies showed that items and options of each SCD measurement method were quite different. Among those methods, memory-related items predominated, followed by executive function and attention [26]. In the present study, we used 9-items subjective cognitive decline questionnaire (SCD-Q9) which included two dimensions of overall memory function and time comparison, daily activity ability to measure the symptoms of SCD [27]. Previous study found that subjective cognitive complaints were associated with greater global brain atrophy and smaller total intracranial volume among people aged over 60 years. And the weaker the people’s daily activity ability, the more tightly the association [28].

This study aims to explore the socio-demographic characteristics of older adults with SCD symptoms and the relationship between SCD symptoms and common chronic diseases, which will benefit health systems to focus on higher risk groups and intervening cognitive decline in Chinese older adults. We hypothesize that SCD symptoms, including decreased memory function and daily activity ability are related to common chronic physical disease. Poor sleep quality, depression symptoms, anxiety symptoms could be the risk factors of worse SCD symptoms.

## Methods

### Study design and participants

A cross-sectional study was conducted from November 2020 to March 2021 in Guangzhou, Guangdong province, China. A total of 789 participants have accepted face-to-face interviews in 7 communities and 2 nursing homes. Among the participants, we excluded those who had neurological diseases (eg. Parkinson’s syndrome, epilepsy, Alzheimer’s), cerebrovascular disease (eg. stroke, cerebral infarction), common mental health diseases (sleep disorders, major depressive disorder, anxiety disorder), and those who were too ill to take part in the survey. Finally, a total of 688 (87.20%) participants were included in the analysis.

This study was in accordance with the Declaration of Helsinki and approved by the Research Ethics Committee of the Guangdong Provincial People’s Hospital, Guangdong Academy of Medical Sciences (No: GDREC2018543H (R1)). All participants provided handwritten informed consent to take part in the study.

### Data collection

Face-to-face interviews with structured questionnaires were conducted to collect information at local communities or nursing homes. All information was entered into the database through questionnaire star, which is an electronic questionnaire system.

### Measurement of subjective cognitive decline symptoms

We used the 9-items subjective cognitive decline questionnaire (SCD-Q9) to measure the symptoms of SCD. The SCD-Q9 is a simple and quick screening scale to identify those who suffer from MCI from general populations [27, 29]. SCD-Q9 lists the 9 core items of SCD symptoms and contains 2 dimensions and 9 items, including the overall memory function and time comparison (4 items), daily activity ability (5 items), with each item’s score may be 0, 0.5 or 1. Total score of SCD-Q9 distributes from 0 to 9, with higher score representing worse SCD symptoms [30]. SCD-Q9 showed good internal reliability and validity among older adults in northern China, and Cronbach’s alpha was equal to 0.886 [27]. The items included in SCD-Q9 were introduced in Table 1. In this study, Cronbach’s alpha was 0.78 for the SCD subscale. According to the cut-off value recommended by Hao et al. and the median (4 points) of the SCD score of our participants, we defined the participants into two groups, with a score less than or equal to 3 as a low score group and a score greater than 3 as a high score group [31].

**Table 1:** Distribution of SCD-Q9 score in the participants [n(%)]

SCD-Q9 items	No/Never (0 point)	Occasionally (0.5 points)	Yes/Often (1 points)
1.Do you think you have problems with your memory?	258 (37.5)	-	430 (62.5)
2.Do you have difficulty remembering a conversation from a few days ago?	399 (58.0)	-	289 (42.0)
3.Do you have complaints about your memory in the last 2 years?	268 (39.0)	-	420 (61.0)
4.How often is the following a problem for you: Personal dates (e.g., birthdays)?	602 (87.5)	59 (8.6)	27 (3.9)
5.How often is the following a problem for you: Phone numbers you use frequently?	509 (74.0)	112 (16.3)	67 (9.3)
6.On a whole, do you think that you have problems remembering things that you want to do or say?	391 (56.8)	-	297 (43.2)
7.How often is the following a problem for you: Going to the store and forgetting what you wanted to buy?	337 (49.0)	308 (44.8)	43 (6.3)
8.Do you think that your memory is worse than 5 years ago?	162 (23.5)	-	526 (76.5)
9.Do you feel you are forgetting where things were placed?	397 (57.7)	-	291 (42.3)

*SCD-Q9* 9-items subjective cognitive decline questionnaire

### Measurement of chronic diseases

Information on chronic diseases was obtained by asking as follows: Have you ever been told by a doctor or other medical professionals that you have suffered from the following chronic diseases in the past year? Participants answered either “yes” or “no”. The chronic diseases list was designed on the basis of the International Classification of Disease, 10th Revision (ICD-10). According to previous epidemiological studies and prevalence, we singled out the following chronic diseases and symptoms to analyze their relationship with SCD symptoms: hypertension, diabetes, ischemic heart disease, hyperlipidemia, arthritis, chronic low back pain, chronic nephritis, urolithiasis, chronic gastroenteritis, cataract/glaucoma, gout, osteoporosis, poor sleep quality, depression symptoms, and anxiety symptoms.

Poor sleep quality was assessed by the Pittsburgh sleep quality index (PSQI), which total score ranged 0 to 21 points and ≥ 7 scores meaning poor sleep quality [32]. Patient Health Questionnaire-9 (PHQ-9) and Generalized Anxiety Disorder-7 (GAD-7) were used to measure depression symptoms and anxiety symptoms, the total score equal to 27 and 21 points respectively [33]. Overall score of PHQ-9 ≥ 5 represented having depression symptoms [34]. Overall score of GAD-7 ≥ 5 suggested having anxiety symptoms [35].

### Socio-demographic and lifestyle factors

Information about socio-demographic and lifestyle factors were self-reported by participants, which included gender (male/female), age, education level, marital status, monthly income, residence, current smoking, alcohol drinking, tea-drinking habits, napping habits, physical exercise frequency and Body Mass Index (BMI).

The age of participants was classified as less than or equal to 70 years old (≤ 70) and more than 70 years old (> 70) [36]. Residence was classified as nursing home and community. The education level was assessed into primary school or lower, junior high school, senior high school, and college or higher. Marital status showed whether the participants were currently widowed/separated/single or married/cohabitation. Income was classified into three groups: < 3500 ¥, 3500–5999 ¥, and ≥ 6000 ¥. Current smokers referred to those currently smoking at least 1 cigarette a day and continuing to smoke for 6 months or more. Alcohol drinking meant drinking at least once per week. Tea-drinking habits referred to drinking tea at least four times a week, including green tea, oolong tea, and black tea. Physical exercise frequency was divided into three groups: hardly or never exercise, occasionally exercise (1–2 times a week or less), frequently exercise (3 times a week and more). The napping habits were defined as having napped at least once in the past 7 days [37]. BMI was equal to weight/height^2^ (kg/m^2^). According to the reference standard of the Chinese population, BMI was listed as three groups: < 18.5 kg/m^2^, 18.5–24 kg/m^2^, and ≥ 24 kg/m^2^ [38].

More details of the survey setting can be found in other survey reports [39].

### Statistical analysis

All analysis was operated using SPSS version 26.0 (IBM). Descriptive statistics were used to describe the distribution characteristics of samples. Chi-square tests and multivariate logistic regression analysis were used for exploring the difference of SCD symptoms among older adults with different socio-demographic characteristics and lifestyle factors. Univariate and multivariate logistic regression analysis were applied to explore the correlates of SCD symptoms, including chronic diseases, poor sleep quality, anxiety and depression symptoms. Statistical significance was set at *P* < 0.05.

## Results

A total of 688 participants were included in analysis with the age of 60 years old and over (*Mean* = 73.79, *SD* = 8.28, range: 60–101), while 62.4% of the participants were females. The mean score of the SCD-Q9 was 3.81 ± 2.42, and the median was 4 points. A total of 286 participants (41.6%) were defined as the low score group, while 402 participants (58.4%) were the high score group.

Table 1 showed distribution characteristics of each item score of SCD-Q9.

Table 2 showed the association between SCD symptoms socio-demographic characteristics and lifestyle factors. Multivariate logistic regression analysis revealed that female (*OR* = 1.99, *95%CI:* 1.35–2.93), primary or lower education level (*OR* = 2.58, *95%CI*: 1.38–4.83), nursing home (*OR* = 1.90, *95%CI*: 1.18–3.05), napping habits (*OR* = 1.59, *95%CI*: 1.06–2.40) were independent positive related to high SCD-Q9 score. On the other hand, tea-drinking habits (*OR* = 0.64, *95%CI*: 0.45–0.92), current smoking (*OR* = 0.46, *95%CI*: 0.24–0.90) were independent negative related to high SCD-Q9 score.

**Table 2:** Comparison of socio-demographic characteristics and lifestyle factors between low and high SCD-Q9 score groups (*n*=688)

Variables	Total	Low SCD-Q9 score	High SCD-Q9 score	Univariate analysis^a^		Multivariate logistic regression analysis	
	n(%)	n(%)	n(%)	χ^2^	*P*	*OR (95% CI)*	*P*
Age (years)				20.07	<0.001***		
≤70	285 (41.4)	147 (51.4)	138 (34.3)			1.00	
>70	403 (58.3)	139 (48.6)	264 (65.7)			1.08 (0.73-1.60)	0.699
Female	429 (62.4)	142 (49.7)	287 (71.4)	36.65	<0.001***	1.99 (1.35,2.93)	<0.001***
Widowed/separated/single	241 (35.0)	76 (26.6)	165 (41.0)	15.38	<0.001***	1.07 (0.72-1.58)	0.754
Education level				25.74	<0.001***		
College or higher	116 (16.9)	51 (17.8)	65 (16.2)			1.00	
Senior high school	229 (33.3)	108 (37.8)	121 (30.1)			1.00 (0.66-1.87)	0.693
Junior high school	186 (27.0)	89 (31.1)	97 (24.1)			1.14 (0.66-1.97)	0.643
Primary school or lower	157 (22.8)	38 (13.3)	119 (29.6)			2.58 (1.38-4.83)	0.003**
Monthly income (¥)				15.19	0.001***		
More than 6000	131 (19.0)	35 (12.2)	96 (23.9)			1.00	
3500-5999	414 (60.2)	183 (64.0)	231 (57.5)			0.58 (0.33-1.01)	0.055
Less than 3500	143 (20.8)	68 (23.8)	75 (18.7)			0.44 (0.22-0.85)	0.015*
Residence (Nursing home)	225 (32.7)	54 (18.9)	171 (42.5)	42.49	<0.001***	1.90 (1.18-3.05)	0.008*
Current smoking	65 (9.4)	47 (16.4)	18 (4.5)	27.92	<0.001***	0.46 (0.24-0.90)	0.024*
Alcohol drinking	47 (6.8)	27 (9.4)	20 (5.0)	5.24	0.022*	1.34 (0.66-2.72)	0.422
Tea-drinking habits	271 (39.4)	144 (50.3)	127 (31.6)	24.63	<0.001***	0.64 (0.45-0.92)	0.015*
Napping habits	537 (78.1)	205 (71.7)	332 (82.6)	11.61	0.001***	1.59 (1.06-2.40)	0.026*
Physical exercise frequency				0.78	0.678		
Occasionally exercise	73 (10.6)	33 (11.5)	40 (10.0)			1.00	
Hardly exercise	122 (17.7)	53 (18.5)	69 (17.2)			1.31 (0.69-2.49)	0.415
Frequently exercise	493 (71.7)	200 (69.9)	293 (72.9)			1.15 (0.66-2.00)	0.622
BMI (kg/m^2^)				4.10	0.129		
18.5 to 24	356 (51.7)	155 (54.2)	201 (50.0)			1.00	
< 18.5	55 (8.0)	16 (5.6)	39 (9.7)			1.87 (0.94-6.72)	0.076
≥ 24	277 (40.3)	115 (40.2)	162 (40.3)			1.23 (0.86-1.75)	0.252

^a^Chi-square tests.

*P ≤0.05, **P ≤ 0.01, ***P ≤ 0.001.

*SCD-Q9* 9-items subjective cognitive decline questionnaire

Table 3 showed the association between SCD symptoms and chronic diseases. Multivariate logistic regression analysis suggested that urolithiasis (*OR* = 2.72, *95%CI*: 1.15–6.40), gout (*OR* = 2.12, *95%CI*: 1.14–3.93), poor sleep quality (*OR* = 1.93, *95%CI*: 1.38–2.71), depression symptoms (*OR* = 3.01, *95%CI*: 1.70–5.34) and anxiety symptoms (*OR* = 3.11, *95%CI*: 1.29–7.46) were independent positive related to high SCD-Q9 score.

**Table 3:** Comparison of chronic diseases and mental health between low and high SCD-Q9 score groups (*n*=688)

Chronic diseases	Total	Low SCD-Q9 score	High SCD-Q9 score	Univariate logistic regression analysis ^b^		Multivariate logistic regression analysis^c^	
	n(%)	n(%)	n(%)	*OR (95% CI)*	*P*	*OR (95% CI)*	*P*
Hypertension	394 (57.3)	155 (54.2)	239 (59.5)	1.24 (0.91-1.68)	0.170	1.14 (0.81-1.60)	0.454
Diabetes	153 (22.2)	60 (21.0)	93 (23.1)	1.13 (0.79-1.64)	0.503	1.12 (0.74-1.69)	0.594
Ischemic heart disease	146 (21.2)	46 (16.1)	100 (24.9)	1.73 (1.17-2.55)	0.006**	1.35 (0.87-2.09)	0.184
Hyperlipidemia	149 (21.7)	59 (20.6)	90 (22.4)	1.11 (0.77-1.61)	0.581	1.38 (0.92-2.09)	0.121
Arthritis	111 (16.1)	39 (13.6)	72 (17.9)	1.38 (0.91-2.11)	0.134	1.19 (0.75-1.88)	0.456
Chronic low back pain	172 (25.0)	61 (21.3)	111 (27.6)	1.41 (0.98-2.01)	0.061	1.41 (0.95-2.10)	0.088
Chronic nephritis	42 (6.1)	18 (6.3)	24 (6.0)	0.95 (0.50-1.78)	0.861	1.25 (0.63-2.50)	0.516
Urolithiasis	34 (4.9)	9 (3.1)	25 (6.2)	2.04 (0.94-4.44)	0.072	2.72 (1.15-6.40)	0.022*
Chronic gastroenteritis/peptic ulcer	67 (9.7)	20 (7.0)	47 (11.7)	1.76 (1.02-3.04)	0.043*	1.46 (0.80-2.66)	0.214
Cataract/glaucoma	116 (16.9)	45 (15.7)	71 (17.1)	1.15 (0.76-1.73)	0.506	1.27 (0.81-1.99)	0.300
Gout	61 (8.9)	20 (7.0)	41 (10.2)	1.51 (0.87-2.64)	0.147	2.12 (1.14-3.93)	0.017*
Osteoporosis	100 (14.5)	27 (9.4)	73 (18.2)	2.13 (1.33-3.41)	<0.002*	1.40 (0.84-2.33)	0.199
Poor sleep quality	349 (50.7)	110 (38.5)	239 (59.5)	2.35 (1.72-3.20)	<0.001*	1.93 (1.38-2.71)	<0.001***
Depression symptoms	95 (13.8)	18 (6.3)	77 (19.2)	3.53 (2.06-6.04)	<0.001*	3.01 (1.70-5.34)	<0.001***
Anxiety symptoms	35 (5.1)	7 (2.4)	28 (7.0)	2.98 (1.29-6.93)	<0.001*	3.11 (1.29-7.46)	<0.001***

^b^No adjustment of other variables.

^c^Age, gender, residence, education level, income, current smoking, tea-drinking habits and napping habits were adjusted.

*P ≤0.05, **P ≤ 0.01, ***P ≤ 0.001.

*SCD-Q9* 9-items subjective cognitive decline questionnaire

## Discussion

Since the definition and diagnostic criteria of SCD were just unified in 2014, there were relatively few studies about the distribution and correlates of it [1]. This study explored the profile of SCD symptoms and their correlates such as chronic disease and mental health in the southern Chinese elderly population. We found that female, primary or lower education level, nursing home, napping habits, urolithiasis, gout, poor sleep quality, depression symptoms and anxiety symptoms were independent positive related to worse SCD symptoms. And tea-drinking habits, current smoking were negative associated with worse SCD symptoms. These findings could be of special relevance for the general practice in order to screen for individuals at risk.

In our study, socio-demographic factors and lifestyle were closely linked to SCD symptoms. Female, primary or lower education level, nursing home and napping habits were positive related to SCD symptoms. Karin et al. said that females were more concerned about health than males, and more sensitive to physical changes, the evolution of symptoms so that females had better perception of symptoms and diseases [40]. Therefore, female complaints were significant information despite no positive results in objective tests. Those older adults with widowed/separated marital status were more susceptible to the clinical progression of AD and performed poor performance in all domains of cognition [41, 42]. Older adults in nursing homes would experience greater cognitive decline than older people living in the community [43]. Those results may be related to the physical and psychological health of older adults living in nursing homes. Older adults with lower education would consider poor cognition and express more concerns about it [30]. Additionally, lower education level was significantly related to difficulties in instrumental activities of daily life among cognitively unimpaired older adults [44]. Our study showed the same results as them. For the prevention and early intervention of cognitive impairment related diseases, we should pay more attention to older adults with a low education level. Napping was associated with worse SCD symptoms. Napping benefited memory consolidation, but there are also contradictory opinions that napping was the consequences of brain damage and degeneration and closely related to diseases in older adults [45]. Further research is needed to find the reasons behind napping. Tea-drinking and current smoking were negative associated with worse SCD symptoms. Previous review had shown that tea-drinking has a protective effect on cognitive decline in older adults, which was similar to our results. The caffeine in tea may improve human cognition and catechins contained in tea were strong antioxidants against oxidative stress [46]. Smoking played a protective role in working memory, executive function because of its short-term actions on the cholinergic system [47]. But there were also some standpoints that smoking tightly related to faster decline in verbal memory and processing speed and this relationship was age-dependent and mediated by genotype status of APOE [48, 49]. There may be survivor bias in our participants.

The strong association between urolithiasis and gout and SCD symptoms might be interpreted from the aspect of daily activity ability function. Urolithiasis and gout would obviously cause significant pain and suffering, leading to complaints of the function of daily activity ability and poor life quality among older adults [50]. In addition, gout may be the risk factor of cognitive impairment. Previous studying results about gout and cognitive decline were controversial. Gout was conducted with chronic hyperuricemia. Oxidative stress is also the main cause of cognitive decline-related diseases. The conflicting results were mainly based on two hypotheses that hyperuricemia could reduce the risk of cognitive impairment-related diseases by changing the level of oxidative stress in the brain. And hyperuricemia induces pro-inflammatory and pro-oxidative reactions in the brain leading to cognitive impairment [51]. We cannot conclude the real influence of hyperuricemia on cognitive impairment in our study. But we considered that hyperuricemia may be the risk factor of worse SCD symptoms and cognitive impairment.

In addition to physical diseases, mental health was also related to SCD symptoms. Depression, anxiety and sleep disorders were common in people suffering from dementia and MCI. But there were no clear neurobiological mechanisms to explain their relationships. Our results showed strong associations between SCD symptoms and poor sleep quality, SCD symptoms and depression symptoms, SCD symptoms and anxiety symptoms. In line with previous study, poor sleep quality, depression symptoms, anxiety symptoms were strong associated with worse SCD symptoms [28]. A review demonstrated that depression was related to higher risk of dementia and a prodromal symptom of dementia [52]. The exact role of depression symptomatology in individuals with SCD was unclear. The mechanisms linking cognitive decline with depression are complex. Similar to aging, depression has been found to induce cognitive decline by increasing inflammation, amyloid deposition and neurofibrillary formation, each of which may lead to impairment of neuroplasticity and hippocampus [53, 54]. In addition, depression has a substantial impact on decreasing the ability to live independently and is the principal contributor to disability in the world [55]. When anxiety symptoms and SCD symptoms occur together, people were at increased risk of neurocognitive diseases [23]. For those with poor sleep quality, they tend to have worse SCD symptoms. The impact of poor sleep quality on cognition function could be explained from many aspects. The sleep-wake cycle was regulated by complex interactions among brain regions and neurotransmitter systems, many of which are implicated in memory and cognitive function [55]. From the perspective of cognitive function performance, people with poor sleep quality reported worse abilities about working memory, attentional set shifting, and abstract problem solving [56]. And poor sleep quality also may result in impairment of neuroplasticity by promoting inflammation and disrupting neurogenesis, which will influence cognition function on learning and memory [57, 58]. The mechanisms for anxiety, depression symptoms and sleep quality affecting SCD are complex. From a symptomatic point, anxiety symptoms, depression symptoms and sleep quality should be seen as independent risk factor of worse SCD symptoms.

There were several limitations in this study. First, as with other cross-sectional studies, we cannot establish causality between SCD and chronic diseases and socio-demographic factors. Secondly, chronic diseases were self-reported by participants and not confirmed by medical professionals again, leading toward potential memory bias. However, it was not feasible to use professional clinical facilities to diagnose chronic diseases in such a large sample of epidemiological investigations. Thirdly, although gender was taken into consideration as covariate in all models, it was possible that our sample may not be representative of the broader population because of its biases (62.4% were females). Fourthly, we only studied the correlation of SCD symptoms among people over 60 years old. Previous study also showed that cognitive complaints under 60 years old may also be related to pathological changes in the brain. Further studies need to verify the finding [28]. Fifthly, it was an exploratory study focusing on SCD symptoms but not SCD diagnosis because of the lack of other cognitive-related data and the sensitivity (68.8%) and specificity (85.7%) of the diagnosis of SCD when the cut-off value of SCD-Q9 score was 3 points [31]. Sixthly, our participants only came from one district of a Chinese city and could not represent all the southern Chinese. Despite the above limitations, our study could provide some information about the association between SCD symptoms and common chronic diseases, socio-demographic factors and lifestyle in southern China older adults.

## Conclusions

Worse SCD symptoms were closely related to socio-demographical factors, lifestyle, common chronic diseases and symptoms among southern Chinese older adults. Disease managers can regulate those diseases and factors to early intervention and management for SCD symptoms among southern Chinese older adults.

## Data Availability

The datasets used during the current study are available from the corresponding author on reasonable request.

## Abbreviations

SCD: Subjective Cognitive Decline
MCI: Mild Cognitive Decline
AD: Alzheimer’s Disease
SCD-Q9: 9-items subjective cognitive decline questionnaire
PSQI: Pittsburgh sleep quality index
PHQ-9: Patient Health Questionnaire-9
GAD-7: Generalized Anxiety Disorder-7
ICD-10: International Classification of Disease, 10th Revision
BMI: Body Mass Index
